# Psychological characteristics of caregivers of pediatric patients with chronic rheumatic disease in relation to treatment adherence

**DOI:** 10.1186/s12969-018-0280-7

**Published:** 2018-10-12

**Authors:** Livia de Freitas Keppeke, Juliana Molina, Vanessa Bugni Miotto e Silva, Maria Teresa de Sande e Lemos Ramos Ascensão Terreri, Gerson Dierley Keppeke, Teresa Helena Schoen, Claudio Arnaldo Len

**Affiliations:** 1 Pediatric Rheumatology Unit, Allergy, Immunology and Rheumatology Division, Department of Pediatrics, Universidade Federal de São Paulo/Escola Paulista de Medicina (UNIFESP/EPM), Borges Lagoa Street, 802, Vila Clementino, São Paulo, SP 04083-001 Brazil; 2 Rheumatology Division, Department of Medicine, Universidade Federal de São Paulo/Escola Paulista de Medicina (UNIFESP/EPM), Pedro de Toledo Street, 720, Vila Clementino, São Paulo, SP 04039-002 Brazil; 3 Adolescent Medicine Unit, Department of Pediatrics, Universidade Federal de São Paulo/Escola Paulista de Medicina (UNIFESP/EPM), Botucatu Street, 715, Vila Clementino, São Paulo, SP 04023-062 Brazil

**Keywords:** Medication adherence, Rheumatic diseases, Caregivers/psychology, Pediatrics

## Abstract

**Background:**

Adherence to treatment for chronic diseases is lower in children than in adults, less extensively studied in children and is associated with multiple related factors. The aim of this study is to perform a descriptive analysis of psycho-cognitive aspects of primary caregivers of pediatric patients with chronic rheumatic diseases, as well as socioeconomic and clinical factors, family functioning and treatment satisfaction.

**Methods:**

Primary caregivers of 90 patients were included. Pairs (caregiver plus patient) were grouped as presenting good adherence (*n* = 50) or poor adherence (*n* = 40) according to the Morisky Adherence Test. Psycho-cognitive aspects were evaluated by Adult Self-Report and Wechsler Adult Intelligence Scale tests. For statistical comparisons, quantitative variables with normal distribution were analyzed by Student’s t test, and those with non-Gaussian distribution with the Mann Whitney test. Categorical variables were analyzed by Chi square test. A multivariate logistic regression analysis was performed to estimate the contribution of the independent variables to adherence.

**Results:**

Compared to caregivers in the good adherence group, caregivers in the poor adherence group were more likely to be classified as clinical on the scales for attention problems and externalizing problems, which include impulsiveness and aggressiveness. They also scored higher on the depressive problem scale. In addition, the average number of children per caregiver and the mean age of caregivers and patients were significantly higher in the poor adherence group, while the proportion of caregivers with higher education was lower. The poor adherence group also included a higher incidence of pediatric patients assuming sole responsibility for managing medications. Economic status, clinical factors, treatment satisfaction, family functioning and caregiver cognitive profile were not related to adherence, except for working memory index.

**Conclusion:**

Older patients, patients as the one solely responsible for medication management, and caregivers with externalizing problems, were observed to be the most strongly associated to poor adherence. Interventions aimed at adolescent patients are needed. Also, psychological programs and interventional studies to better determine caregivers’ behavioral/emotional status, and parent-child relationships are recommended.

## Background

Childhood rheumatic diseases have significant physical, mental, emotional, economic and social impact on patients and their families [1]. Treatment regimens for these diseases are complex and require constant adherence for a long period of time in order for beneficial effects to be observed and unwanted side effects to be minimized. These factors increase the risk of poor adherence [2]. In addition to pharmacological treatment, appropriate physiotherapy conditioning, nutritional support and psychosocial assistance are often required [1].

Adherence is defined by the World Health Organization as the extent to which a person’s behavior in taking medication, following a diet, or making lifestyle changes corresponds to the recommendations of a health care provider [3]. Adherence to treatment for chronic diseases is lower in children than in adults and is less extensively studied in children [4].

Poor adherence in children is associated with multiple related factors, including social, demographic, cultural, and psychological factors, and depends on the triad relationship between family, patient and health care provider. Satisfaction with treatment, understanding of disease and treatment by patients and their caregivers, complexity of the disease and/or the treatment plan and the health care system adopted may also interfere with adherence [5–8].

Regarding socio-economic factors, studies have shown that limited financial resources and low education of caregivers are associated with health problems and poor adherence to treatment regimens in children [9, 10]. The authors point out that part of the difficulty in adapting to the prescribed regimen may be due to stress related to low socioeconomic status and poor social support [11, 12].

Concerning clinical factors, disease severity at the time of diagnosis proves to be a decisive factor for good adherence. Moreover, medication regimens involving no more than two daily medications, followed by rapid beneficial effects and few side effects, facilitate adherence [5, 13].

Regarding family factors, high rates of adherence are observed among patients who belong to cohesive families that include caregivers who are in a stable relationship [13], have good social skills such as assertiveness and empathy when interacting with children [14, 15] and support the socialization of children with friends [16].

Remembering to take medications as instructed, despite distractions inherent to busy lifestyles, is a behavior dependent on the ability to plan and execute multiple tasks simultaneously and/or sequentially, which requires cognitive skills such as attention, memory and executive functions [17, 18]. Few studies have been done to evaluate the association between treatment adherence and cognitive functioning. Most of the existing studies were conducted in adult patients with systemic lupus erythematosus (SLE) or acquired immune deficiency syndrome (AIDS) [17, 19–22]. The number of studies on treatment adherence related to cognitive abilities of pediatric caregivers is even scarcer. In one study involving pediatric patients with AIDS, a relationship between low cognitive performance of caregivers and poor adherence was observed, although this relationship declined in multivariate analysis [23].

Considering that socioeconomics, clinical care, family and emotional / behavioral factors are known to influence adherence to treatment of chronic disease, and that little or no knowledge is available about the influence of cognitive aspects of caregivers on adherence in pediatric rheumatology, the aim of this study is to perform a descriptive analysis of psycho-cognitive aspects of primary caregivers of pediatric patients with chronic rheumatic diseases, as well as socioeconomic and demographic status, and family functioning, and to evaluate relationships between these factors and adherence to treatment regimens. We also evaluated the impact of factors related to the disease, the treatment, and treatment satisfaction on treatment adherence.

## Methods

### Participants

This cross-sectional study included primary caregivers of 90 pediatric patients previously diagnosed with juvenile idiopathic arthritis (JIA; *n* = 37), juvenile dermatomyositis (JDM; *n* = 16) or juvenile systemic lupus erythematosus (JSLE; *n* = 37), according to international criteria [24–27]. The participant pairs (caregiver plus patient) were recruited between 2013 and 2015 from the Pediatric Rheumatology Clinic of the São Paulo’s Federal University Hospital (UNIFESP/HSP) and were followed up for at least six months with a minimum of one immunosuppressive and/or biological agent prescription.

Participants were grouped as presenting good adherence or poor adherence according to the Morisky, Green and Levine Medication Adherence Test (see below) [28]. In a pre-selection, the adherence test was given to 146 pairs of caregivers and patients, all caregivers and the patients older than 10 years (*n* = 63) answered the test. However only adherence test results for the respondent responsible for managing medications, which could be the caregiver, the patient or the patient assisted by caregiver (Table 2), were used to determine good/poor adherence status. Caregivers with conditions that could affect the Wechsler Adult Intelligence Scale (WAIS-III) test results (hearing and visual impairment, brain injury and other neurological damage) were not included. The study was approved by the Ethics Committee of UNIFESP (Process number CEP 262.434).

### Procedure

Caregivers and their children that met the inclusion criteria were randomly approached and asked to participate in the study while waiting for their scheduled medical appointments. Upon agreement, the participants, caregivers and patients, gave their consent for the study by signing the informed consent or assent forms. After the medical consultation, they were sent to a quiet room where they completed the various study instruments. Each respondent answered the instruments in a single session that lasted around two hours, always with the same researcher (the first author).

### Measures

#### Morisky, green and Levine test [28]

This is a four-item generic self-reported medication-taking behavior scale. The items were: 1- Do you ever forget to give the medicine to your child? 2- Are you careless at times about giving the medicine to your child? 3- When your child feels better do you sometimes stop giving him the medicine? 4- Sometimes your child feels worse, when he/she takes the medicine, do you stop giving the medicine to him/her? One or more “yes” answers indicates poor adherence. Previous studies have shown the usefulness of this test, which has been translated into Portuguese and validated in Brazil [29, 30].

#### Demographic chart

This questionnaire was developed specifically for this study in order to collect sociodemographic information (age, gender, schooling, family composition, responsibility for management of medications). Due to low representation of certain educational levels in the study sample, educational levels were grouped into two stages: “Stage 1” including those who had completed any number of years of elementary school education, and “Stage 2” including those who had completed high school and higher education. Data was collected during interviews with participants. Clinical data (diagnosis, follow-up at the clinic, disease activity, quantity of prescribed medications) was collected by analyzing medical charts.

#### Brazil standard criteria for economic classification 2012

Published by Associação Brasileira de Empresas de Pesquisa (ABEP), this scale defines socioeconomic level based on the purchasing power of households and the degree of education of the head of the household. The higher the total score, the higher the economic class. Available from: <http://www.abep.org/criterio-brasil>.

#### Pediatric quality of life inventory - healthcare satisfaction generic module 3.0 (PedsQL-SSS)

This is a self-administered survey for parents of pediatric patients with chronic diseases to measure of satisfaction with health care [31, 32].

#### Family APGAR scale [33, 34]

This screening test gives a rapid overview of family function. It is designed to permit measurement of family members’ satisfaction with each of five basic components of family function: adaptation, partnership, growth, affection and resolve. Depending on the score, the quality of family function is characterized as “severely dysfunctional”, “moderately dysfunctional” or “highly functional”. For analysis of family functioning, we did not include grandmothers and aunts who acted as caregivers (*n* = 5) (Table 2), because even though they live with and take care of the pediatric patient, their role in the family does not correspond to that of parents.

#### Adult self-report (ASR) [35]

This instrument was used to assess behavioral/emotional problems in caregivers. It is divided into scales that assess syndromes (such as internalizing problems, externalizing problems, and attention problems), adaptive functioning, substance use, and Diagnostic and Statistical Manual of Mental Disorders-Oriented scales (such as depressive problems, and anxiety problems) (DSM-V). Each scale has specific cutoff points which correspond to normal, borderline or clinical. This instrument demonstrated satisfactory evidence of reliability [36]. In our study, we evaluated attention problems, depressive problems and anxiety problems, as well as internalizing problems, externalizing problems and total problems (internalizing problems plus externalizing problems).

#### Wechsler adult intelligence scale (WAIS-III) – Short form 8 (SF8) [37]

SF8 was validated in Brazil for research purposes [38]. The test is comprised of verbal and executive subtests. A participant’s performance may be characterized in a general way, with results expressed as Total IQ, Verbal IQ and Executive IQ, or in a more specific way, with results of the factorial indexes (verbal comprehension index, perceptual organization index, working memory index and processing speed index) reported in addition to the full scale results.

### Statistical analysis

Shapiro-Wilk test was employed to assess the normality of the data. Quantitative variables with normal distribution were analyzed by Student’s unpaired t test, and those with non-Gaussian distribution were analyzed using the Mann Whitney test. Categorical variables were analyzed by Chi square test adding Bonferroni correction when three or more groups were compared.

A multivariate logistic regression analysis was performed to estimate the independent value of the psycho-cognitive and sociodemographic characteristics on poor adherence. Due to the large number of predictor variables, we selected for the initial model variables with significant association at 10% in the univariate analysis. For the predictor variables that were present in the quantitative and categorical form, we considered the categorical form in order to facilitate the interpretation of results. The non-significant variables at 5% were excluded one-by-one by order of significance (backwards method) to reach the final model. The final model adequacy was evaluated by the Hosmer and Lemeshow test.

All data was analyzed using SPSS 19.0 or GraphPad Prism 5.0 for Windows. The data is presented as proportions (%), adjusted odds ratio (OD), or averages plus standard deviation (±), unless stated otherwise. Values of *p* ≤ 0.05 were considered statistically significant.

## Results

From June 2013 to April 2015, 146 pairs of caregivers and patients were screened with the medication adherence scale. Of these, five who would be classified in the poor adherence group did not agree to participate in the study and the last 51 to be evaluated that would be classified in the good adherence group were not included because the study required participants presenting poor adherence. Thus, among the 146 who participated in the pre-selection, 45 (30.8%) presented poor adherence, while 101 (69.2%) presented good adherence.

The final sample consisted of 90 primary caregivers and patients older than 10 years (*n* = 63) were also evaluated. Forty (44.5%) participants were classified in the poor adherence group and 50 (55.5%) in the good adherence group. In the poor adherence group, 19 (47.5%) presented unintentional failure, 3 (7.5%) presented intentional failure, and 18 (45%) presented the two forms of failure.

For patients, the mean age of those in the poor adherence group was higher than that of those in the good adherence group (14.1 vs 10.1 yrs.; *p* < 0.0001; Mann Whitney Test). Time of follow-up, disease activity and quantity of prescribed medications were similar in poor and good adherence groups (Table 1).

**Table 1:** Distribution of sociodemographic and clinical characteristics and treatment satisfaction in relation to good and poor adherence

			Total (*n* = 90)	Good Adherence 55.5% (50)	Poor Adherence 44.5% (40)	*p*
Patient	Age		11.9 (±4.4)	10.1 (±4.3)	14.1 (±3.4)	< 0.0001***
	Follow-up (years)		3.7 (±3.7)	3.1 (±3.0)	4.4 (±4.4)	0.150
	Disease activity	Active	52.8% (47)	52% (26)	53.8% (21)	0.516
		Inactive	47.2% (42)	48% (24)	46.2% (18)	
	Quantity of prescribed medications		5.5 (±2.2)	5.2 (±2.2)	5.8 (±2.2)	0.294
Caregiver	Age		39.2 (±8.6)	37.1 (±8.2)	41.7 (±8.5)	0.0113*
	Kinship	Mother	82.2% (74)	86% (43)	77.5% (31)	0.252
		Father	12.2% (11)	12% (6)	12.5% (5)	
		Other ^a^	5.6% (5)	2% (1)	10% (4)	
	Marital status	Married	75.5% (68)	82% (41)	67.5% (27)	0.090
		Other ^b^	24.5% (22)	18% (9)	32.5% (13)	
	Schooling	Stage 1 ^c^	47.8% (43)	38% (19)	60% (24)	0.031*
		Stage 2 ^d^	52.2% (47)	62% (31)	40% (16)	
	ABEP socioeconomic score		20.9 (±5.2)	21.1 (±4.6)	20.6 (±5.9)	0.713
	Treatment satisfaction			90.1 (±11.0)	91.5 (±9.3)	0.471

^a^grandmother or aunt; ^b^ widowed, single or divorced; ^c^ incomplete and complete elementary school; ^d^ completed high school and complete higher education

*p < 0.05; ***p < 0.001

For caregivers, the mean age of those in the poor adherence group was also higher than mean age of those in the good adherence group (41.7 vs 37.1 yrs.; *p* = 0.011; unpaired t test). A smaller proportion of caregivers with higher education level was observed in the poor adherence group compared to the good adherence group (40% vs 62%; *p* = 0.031; Chi square test). Sociodemographic factors and treatment satisfaction did not differ significantly between the caregivers in the poor and good adherence groups (Table 1).

Regarding family functioning, the average scores on the Family APGAR scale were similar between good and poor adherence groups. For categorical analysis we classified the scores as “Highly Functional” or “Dysfunctional”, combining “Moderately Dysfunctional” and “Severely Dysfunctional” into the “Dysfunctional” category due to low representation of those classifications. The categorical analysis showed a higher proportion of “Dysfunctional” in the poor adherence group and higher proportion of “Highly Functional” in the good adherence group, although these differences did not reach statistical significance (Table 2).

**Table 2:** Distribution of family functioning, number of children, and responsibility for medication management in relation to good and poor adherence

		Good Adherence (^a^n=49/^b^n = 50)	Poor Adherence (^a^n=36/^b^n = 40)	*p*
Family Functioning ^a^	Highly Functional	71.4% (35)	58.3% (21)	0.152
	Dysfunctional	28.6% (14)	41.7% (15)	
	Average score	14.9 (±4.6)	13.2 (±5.3)	0.118
Number of Children ^b^		2.1 (±0.8)	2.9 (±1.8)	0.004**
Responsibility for Medication Management ^b^	Caregiver	66% (33)	35% (14)	< 0.0015**
	Patient	6% (3)	47.5% (19)	
	Patient and Caregiver	28% (14)	17.5% (7)	

^a^sample without grandmothers and aunts; ^b^ sample with grandmothers and aunts

**p < 0.01

We also analyzed the number of children and responsibility for medication management in relation to adherence. The average number of children per caregiver was higher in the poor adherence group compared to the good adherence group (2.9 vs 2.1; *p* = 0.004; Mann Whitney Test). The proportion of families where the patient was solely responsible for medication management was higher in the poor adherence group compared to good adherence group (47.5% vs 6%; *p* < 0.0015; Chi square test with Bonferroni adjustment), while the proportion of families where the caregiver or the patient together with the caregiver were responsible for medication management was higher in the good adherence group compared to the poor adherence group (66% vs 35% and 28% vs 17.5%, respectively; p < 0.0015; Chi square test with Bonferroni adjustment) (Table 2).

When caregivers’ behavioral profiles were analyzed we found that, compared to caregivers in the good adherence group, those in the poor adherence group scored significantly higher on the depressive problems (*p* = 0.028; Mann Whitney test), attention problems (*p* = 0.0003; Mann Whitney test), externalizing problems (*p* = 0.013; unpaired t test), and total problems (*p* = 0.0017; Mann Whitney test) scales. Those in the poor adherence group also scored higher on the internalizing problems scale, but the difference did not quite reach statistical significance (*p* = 0.063; unpaired t test) (Table 3).

**Table 3:** Caregivers’ behavioral/emotional profile in relation to good and poor adherence

		Mean (± SD)	*p*	Classification		*p*
				Normal	Clinical	
Depressive Problems	Good Adherence	57.7 ± 8.8	0.028*	74% (37)	26% (13)	0.301
	Poor Adherence	61.3 ± 9.1		66.7% (26)	33.3% (13)	
Anxiety Problems	Good Adherence	62.8 ± 6.9	0.384	62% (31)	38% (19)	0.212
	Poor Adherence	64.2 ± 7.6		51.3% (20)	48.7% (19)	
Attention Problems	Good Adherence	56.0 ± 5.8	0.0003***	88% (44)	12% (6)	0.008**
	Poor Adherence	61.3 ± 7.0		64.1% (25)	35.9% (14)	
Internalizing Problems	Good Adherence	59.4 ± 10.6	0.063	52% (26)	48% (24)	0.096
	Poor Adherence	63.6 ± 10.5		35.9% (14)	64.1% (25)	
Externalizing Problems	Good Adherence	52.8 ± 8.2	0.013*	80% (40)	20% (10)	0.008**
	Poor Adherence	58.2 ± 12.0		53.8% (21)	46.2% (18)	
Total Problems	Good Adherence	54.8 ± 8.9	0.0017**	76% (38)	24% (12)	0.007**
	Poor Adherence	60.9 ± 10.2		48.7% (19)	51.3% (20)	

*p < 0.05; **p < 0.01; ***p < 0.001

For categorical data analysis, we grouped the borderline and clinical classifications into one category termed “clinical” due to low representation in these categories. When these categories were analyzed for each behavioral parameter, we found that, compared to caregivers in the good adherence group, a higher proportion of caregivers in the poor adherence group were classified as clinical with regard to attention problems (35.9% vs 12%; *p* = 0.010), externalizing problems (46.2% vs 20%,; *p* = 0.008) and total problems (51.3% vs 24%; *p* = 0.014; Chi square test) scales, with no significant difference in the other scales. (Table 3). Internalizing problems are dysfunctional private behavior patterns, which are characterized by dysphoria and retreat. Externalizing problems are dysfunctional behaviors against others and the environment, which include opposition, aggression, impatience, mood fluctuations, teasing, and self-centeredness. Total problems are the plus of internalizing and externalizing problems. In summary, caregivers in the poor adherence group scored higher and were more likely to be classified as clinical on the attention problems, externalizing problems and total problems scales. They also scored higher on the depressive problem scale.

Regarding cognitive profiles of caregivers, no significant differences were observed in mean Total IQ, Verbal IQ or Executive IQ between caregivers in the poor and good adherence groups. Similarly, no significant differences were seen in the mean scores for factor indexes (Table 4).

**Table 4:** Caregivers’ cognitive profile in relation to good and poor adherence

		Mean (± SD)	*p*	IQ Classification			*p*
				≤ Below Average (69 to 89)	Average (90 to 109)	≥ Above Average (≥ 110)	
Total IQ	Good Adherence	95.6 ± 9.5	0.827	30% (15)	54% (27)	16% (8)	0.274
	Poor Adherence	96.1 ± 13.6		39.5% (15)	36.8% (14)	23.7% (9)	
Verbal IQ	Good Adherence	92.4 ± 8.5	0.708	40% (20)	56% (28)	4% (2)	0.109
	Poor Adherence	93.2 ± 12.5		50% (19)	36.8% (14)	13.2% (5)	
Executive IQ	Good Adherence	99.8 ± 12.6	0.936	24% (12)	56% (28)	20% (10)	0.836
	Poor Adherence	99.8 ± 14.6		28.9% (11)	50% (19)	21.1% (8)	
Verbal Comprehension Index	Good Adherence	90.1 ± 8.7	0.935	50% (25)	48% (24)	2% (1)	0.403
	Poor Adherence	90.3 ± 12.8		50% (19)	42.1% (16)	7.9% (3)	
Perceptual Organization Index	Good Adherence	96.9 ± 12.5	0.467	32% (16)	52% (26)	16% (8)	0.639
	Poor Adherence	99.5 ± 14.5		31.6% (12)	44.7% (17)	23.7% (9)	
Working Memory Index	Good Adherence	95.5 ± 10.0	0.687	22% (11)	72% (36)	6% (3)	0.080
	Poor Adherence	96.4 ± 14.0		39.5% (15)	44.7% (17)	15.8% (6)	
Processing Speed Index	Good Adherence	108.4 ± 11.4	0.716	4% (2)	56% (28)	40% (20)	0.368
	Poor Adherence	107.4 ± 14.8		10.6% (4)	44.7% (17)	44.7% (17)	

For categorical data analysis, we grouped the IQ results into tertiles defined as below average (69–89), average (90–109) and above average (≥110). The proportion of scores in the below average, average and above average tertiles were determined for Total IQ, Verbal IQ and Executive IQ. No significant difference in tertile proportions between caregivers from the good and poor adherence groups was found for any of the IQ scores. There were trends, however, toward a higher proportion of caregivers scoring below average on the Working Memory Index (WMI) in the poor adherence group compared to the good adherence group (39.5% vs 22%), and a trend for higher proportion of caregivers scoring average on the WMI in the good adherence group compared to the poor adherence group (72% vs 44.7%; *p* = 0.080; Chi Square Test with Bonferroni adjustment) (Table 4). Overall, this data indicates that caregivers cognitive functioning profile as assessed by the WAIS-III test is not associated with medication adherence.

To clarify the independent value of the psycho-cognitive and sociodemographic characteristics, a multivariate logistic regression analysis was performed (Table 5). In the final model, what remains significant is the patient’s age (*p* = 0.026), the patient as solely responsible for managing medication (*p* = 0.008), externalizing problems (*p* = 0.012) and the WMI (*p* = 0.041). Regarding the patient’s age, for each 1 year increase there was a 17.0% (OD = 0.83) reduction in the chance of adherence, adjusted for the presence of the other variables in the model. In addition, the patient’s chance of adherence was 91% lower (OD = 0.09) if he is solely responsible for managing medication than if the caregivers are responsible for administering the medication. There were no differences in chance of adherence among those in whom both the patient together with the caregiver were responsible for medication management and those whose caregivers were solely responsible (OD = 1.28; *p* = 0.729). It was also noted that children whose caregivers were classified as clinical in externalizing problems have 81% (OD = 0.19) lower chance to present good treatment adherence than that of children whose caregivers were classified as normal. In the caregivers WMI, when the index was below average, there was a 66% (OD = 0.34) lower chance of good adherence, or when the index was above the average, curiously there was also an 88% (OD = 0.12) lower chance of good adherence. This result indicates that working memory index within the average favors good adherence to the treatment.

**Table 5:** Multivariate logistic regression analysis on poor adherence

	Initial model		Final model	
	Adjusted OR (CI95%)	*p*	Adjusted OR (CI95%)	*p*
Patient’s age	0.78 (0.63–0.98)	0.032	0.83 (0.71–0.98)	0.026
Caregiver				
Age	1.09 (0.98–1.21)	0.112	–	–
Marital status (ref. = Married)				
Other	0.41 (0.08–2.10)	0.285	–	–
Schooling (ref. = Stage 1)				
Stage 2	3.39 (0.75–15.31)	0.112	–	–
Number of Children	0.70 (0.35–1.37)	0.295		
Responsibility for Medication Management (ref. = Caregiver)		0.008		0.008
Patient	0.042 (0.005–0.355)	0.004	0.09 (0.02–0.49)	0.006
Patient and Caregiver	1.04 (0.19–5.76)	0.966	1.28 (0.32–5.11)	0.729
Depressive Problems - score	1.03 (0.90–1.17)	0.689	–	–
Clinical Attention Problems	0.24 (0.03–1.82)	0.169		
Clinical Internalizing Problems	0.91 (0.13–6.12)	0.919	–	–
Clinical Externalizing Problems	0.20 (0.03–1.29)	0.091	0.19 (0.05–0.69)	0.012
Clinical Total Problems	1.61 (0.17–15.05)	0.678	–	–
Working Memory Index (ref. = Average)		0.056		0.041
≤ Below Average	0.42 (0.08–2.06)	0.283	0.34 (0.09–1.23)	0.099
≥ Above Average	0.07 (0.01–0.63)	0.018	0.12 (0.02–0.76)	0.024

*N* = 88

Hosmer and Lemeshow test for initial model (*p* = 0.510) and final model (*p* = 0.708)

## Discussion

This study is unique in pediatric rheumatology because, in addition to considering socioeconomic, clinical and family factors, we also investigated the association between behavioral and cognitive profiles of primary caregivers in relation to medication adherence.

In the pre-selection phase, we obtained a rate of poor adherence to the medication of 30%. A previous study by our group showed a similar rate of poor adherence, 20.2% [13]. Other studies in rheumatic diseases also observed similar rates of poor adherence, ranging from 15 to 48% [5, 39, 40]. However, we must consider that these rates vary according to the method used. In the present study we used the Morisk, Green and Levine test that is useful to evaluate medication adherence in practically all type of diseases. The previous study used a scale developed by the researchers themselves [41]. Usually more objective evaluation methods, such as electronic monitoring and counting of the number of tablets, detect higher rates of poor adherence in relation to self-reported scales [5, 42, 43]. We chose to use the Morisk, Green and Levine test because it is a brief instrument, easy to apply and has been designed to underestimate good adherence and overestimate poor adherence.

Our study revealed a correlation between poor adherence and caregiver profiles indicative of behavioral problems, but no correlation between poor adherence and caregivers’ cognitive functioning profiles, except for WMI in the multivariate analysis. We also found that caregivers in the poor adherence group had higher mean age and lower education level compared to those in the good adherence group, although these observations do not remain significant in the multivariate analysis. The mean age of pediatric patients in the poor adherence group was also higher, and the multivariate analysis strongly confirmed this finding. This result probably explains why a higher proportion of older caregivers belonged to poor adherence group in the univariate analysis. More patients took the responsibility of managing medications in the poor adherence group than in the good adherence group, and the multivariate analysis also strongly confirmed this finding. In addition, families in the poor adherence group had a significantly higher mean number of children compared to those in the good adherence group; however this association was not observed in the multivariate analysis. Some sociodemographic characteristics such as caregivers with higher mean age, lower education level, and with higher mean number of children were related to poor adherence, but only in the univarietary analysis. Still, we suggest that special attention should be given to caregivers with these characteristics, in order to refer them to a psychological or/and psychiatric consultation when necessary.

The higher mean age of patients in the poor adherence group is consistent with previous studies that indicate an increase in poor adherence in children as they grow older and become adolescents [4, 7, 23, 44]. Several characteristics of adolescents could be considered risk factors for poor adherence. One is a sense of omnipotence [45, 46], which might lead adolescent patients to assume that dangerous situations have no consequences. Hence, they may stop treatment as a way to test whether they need to continue adhering, especially when they are asymptomatic, even if they had experienced serious morbidity previously. Furthermore, adolescents often engage in several extra-curricular activities and generally spend most of their time at school, which may hinder the incorporation of a medication regimen into their routines.

Another characteristic of adolescents that might impact medication adherence is the desire for quick results. Control of chronic rheumatic disease only occurs after a long period of treatment. In the short term the treatment may cause undesirable side effects such as change in appearance and malaise. Adolescents also prefer to make decisions for themselves, which can make it difficult to convince them to follow treatment regimens if they do not want to [47].

We observed a higher prevalence of adherence failures among adolescents who took sole responsibility for administration of medications, as has been seen in previous studies [9, 48]. In general, adolescents do not have fully developed autonomy, and thus lack skills required to assume sole responsibility for following treatment regimens. A study of patients with JIA showed that it is common for adolescents with this chronic disease to have difficulty with the management of the treatment during the transition to adulthood [49]. In this same study it was observed that the most common reason for skipping medications was forgetfulness coupled with difficulty in taking medications as directed, keeping a calendar of appointments, and maintaining a personal medical file. Executive functioning, which includes planning and medications self-management, is among the last functions to be acquired during brain development [50], which is not to say that good training cannot help the adolescent improve medication management. Therefore, adolescent patients who take on the responsibility for medication management might have better treatment adherence if they assume that responsibility gradually under the supervision of adult caregivers.

Furthermore, other reasons can delay the improvement of self-management in adolescent patients. The risk for parental overprotection of children with a chronic disease may delay the process of gaining independence from caregivers. Some transition programs have been running in pediatric rheumatology clinics to provide adolescent-oriented care in order to improve self-management. These programs also aim to promote successful transfer to adult rheumatology care. Some strategies within such programs include parenting orientation. The adolescent patients also have some small amount of time alone during consultation for confidential discussions regarding health issues including sexual health, risk-taking behaviors, family problems, and vocational issues [51].

Caregivers in the poor adherence group showed more behavioral profile problems than those in the good adherence group, specifically in the areas of attention problems, depressive problems and externalizing problems. However, based on the multivariate analysis, we observed that only externalizing problems interfere effectively on medication adherence. Attention problems include problems with forgetfulness, concentration, planning, task completion, setting priorities, lack of energy, disorganization, and a tendency to lose things. These problems can be the result of cognitive impairment, but we did not observe significant differences in cognition between caregivers in the poor and good adherence groups through the univariate analysis. However, it called our attention that in the multivariate analysis, caregivers with WMI that were grouped below the average or above the average presented a lower chance of good adherence. Therefore, the attention problems observed in this study may reflect the difficulty in working memory ability. However, more specific neuropsychological instruments to investigate these aspects would be necessary to confirm this hypothesis. In addition, attention problems experienced by caregivers in the poor adherence group could be related to emotional factors and/or the complexity of life in a big city. Families with individuals inflicted by chronic disease are burdened due to the usual complex treatment regimen, especially when government support is not ideal. Health professionals could help lessen the impact of working memory difficulty and attention problems on treatment adherence by developing programs to help caregivers manage treatment and successfully incorporate treatment regimens into their busy lives.

Caregivers in the poor adherence group also exhibited more depressive problems than those in the good adherence group, according to univariate analysis. Although, multivariate analysis did not confirm depressive characteristics as effectively affecting adherence, special attention should be given to caregivers with such characteristics. Depressive problems include sadness, difficulty making decisions, lack of energy, and pessimism. Caregivers with depressive problems may lack motivation and vitality to accomplish daily activities, including maintaining a medication regimen. Due to lack of emotional resources, caregivers with depressive problems may shift the responsibility for managing medications to the patient, possibly without proper training. Depressive problems characteristics observed in this study may also be a reflection of externalizing problems, which were seen to interfere in adherence and were strongly associated to poor adherence in both univariate and multivariate analysis. Externalizing problems include opposition, aggression, impatience, mood fluctuations, teasing, and self-centeredness. Impulsivity is frequently found in depressed individuals and correlates positively with aggressive behavior [52]. Individuals with major depressive disorder show higher impulsivity and more severe aggression than individuals without these conditions [53].

Those in the poor adherence group also reported more internalizing problems in the univariate analysis, which are characterized by dysphoria and retreat, but the difference was not significant. Other studies have found that caregivers of pediatric patients with chronic diseases often have quality of life issues, including internalizing problems [54–56]. Altogether, these findings underscore the importance of emotional and behavioral status of caregivers in adherence to treatment regimens for children with chronic diseases.

Although adherence to treatment regimens requires cognitive skills, in this study cognitive functioning problems in caregivers was not associated with poor adherence in the univariate analysis. However, we observed a lower chance of good adherence in caregivers with WMI grouped below average and above average. Caregivers with above average WMI presenting lower chance to adherence may result from a coincidence as the total number of caregivers with this condition was low (*n* = 9). In any case, we observed that working memory index within the average favors good adherence to the treatment. We found only one report association between problems in cognitive functioning of caregivers and poor adherence to medication treatment, which was a study performed in children with AIDS [23]. However, that association lost significance in multivariate analysis. Nevertheless, this AIDS study shows the importance of considering the emotional state of caregivers when evaluating cognitive functioning.

In the present study more caregivers in the poor adherence group scored below average on total, verbal and executive IQ, and on WMI and processing speed index compared to those in the good adherence group, but the differences did not reach statistical significance except for WMI in the multivariate analysis. It is known that the working memory is one of the essential cognitive functions that helps in organizing and planning and in facilitating the administration of medications [17, 18]. Since a difficulty in working memory ability, and the presence of attention problems in caregivers in the poor adherence group was observed, more specific neuropsychological instruments to investigate these aspects would be necessary. Our study was not without limitations. Adherence was evaluated only through self-reporting, rather than through more direct and objective methods, such as counting the number of pills or measuring serum levels of medications. The absence of specific neuropsychological instruments to assess attention and working memory, the cross-sectional study design, the relatively small number of study participants, and the variety of illnesses studied were also a limiting factor.

## Conclusions

Our study revealed a correlation between poor treatment adherence and caregivers with behavioral profiles indicative of problems, specifically attention, depressive and externalizing problems. We observed an association between adherence and the cognitive profile of the caregivers in WMI. We also found that caregivers in the poor adherence group were older on average and had a lower level of education compared to those in the good adherence group. Higher number of children per family also was found to be associated with poor adherence. No correlations were found between adherence and family functioning, satisfaction with treatment, clinical data or family economic level.

Older patients, patients as the one solely responsible for medication management, and caregivers with externalizing problems characteristics, were observed to be the most strongly conditions associated to poor adherence. This findings show that interventions aimed at adolescent patients, especially those solely responsible for medication management are needed. We highlight the need for psychosocial monitoring in caregivers of pediatric patients with chronic disease in order to prevent poor adherence, as well as new interventional studies to better determine caregivers’ behavioral/emotional status, and parent-child relationship aiming to promote proper adherence to treatment.

## Abbreviations

ABEP: Associação Brasileira de Empresas de Pesquisa (Research Companies Brazilian Association)
AIDS: Acquired immune deficiency syndrome
ASR: Adult Self-Report
DSM-V: Diagnostic and Statistical Manual of Mental Disorders 5ª edition
IQ: Intelligence quotient
JDM: Juvenile dermatomyositis
JIA: Juvenile idiopathic arthritis
JSLE: Juvenile systemic lupus erythematosus
PedsQL-SSS: Pediatric Quality of Life Inventory - Healthcare Satisfaction Generic Module 3.0
SF8: Short Form 8
SLE: Systemic lupus erythematosus
UNIFESP/HSP: Sao Paulo’s Federal University Hospital
WAIS-III: Wechsler Adult Intelligence Scale 3ª edition
WMI: Working Memory Index

## References

[1] Cassidy JT, Petty RE, Laxer R, Lindsley CB (2011). Textbook of pediatric rheumatology. 6th ed..

[2] Rapoff MA (2006). Management of adherence and chronic rheumatic disease in children and adolescents. Best Pract Res Clin Rheumatol.

[3] WHO (2003). Adherence to long-term therapies: evidence for action.

[4] Costello I, Wong IC, Nunn AJ (2004). A literature review to identify interventions to improve the use of medicines in children. Child Care Health Dev.

[5] Rapoff MA, Belmont JM, Lindsley CB, Olson NY (2005). Electronically monitored adherence to medications by newly diagnosed patients with juvenile rheumatoid arthritis. Arthritis Rheum.

[6] Stepansky MA, Roache CR, Holmbeck GN, Schultz K (2010). Medical adherence in young adolescents with spina bifida: longitudinal associations with family functioning. J Pediatr Psychol.

[7] Pelajo CF, Sgarlat CM, Lopez-Benitez JM, Oliveira SK, Rodrigues MC, Sztajnbok FR (2012). Adherence to methotrexate in juvenile idiopathic arthritis. Rheumatol Int.

[8] Santer M, Ring N, Yardley L, Geraghty AW, Wyke S (2014). Treatment non-adherence in pediatric long-term medical conditions: systematic review and synthesis of qualitative studies of caregivers’ views. BMC Pediatr.

[9] Hsin O, La Greca AM, Valenzuela J, Moine CT, Delamater A (2010). Adherence and glycemic control among Hispanic youth with type 1 diabetes: role of family involvement and acculturation. J Pediatr Psychol.

[10] Keane E, Layte R, Harrington J, Kearney PM, Perry IJ (2012). Measured parental weight status and familial socio-economic status correlates with childhood overweight and obesity at age 9. PLoS One.

[11] Bradley RH, Corwyn RF (2002). Socioeconomic status and child development. Annu Rev Psychol.

[12] Maitra S (2010). Can patient self-management explain the health gradient? Goldman and Smith’s “can patient self-management help explain the SES health gradient?” (2002) revisited. Soc Sci Med.

[13] Bugni VM, Ozaki LS, Okamoto KY, Barbosa CM, Hilario MO, Len CA (2012). Factors associated with adherence to treatment in children and adolescents with chronic rheumatic diseases. J Pediatr.

[14] Bobrow ES, AvRuskin TW, Siller J (1985). Mother-daughter interaction and adherence to diabetes regimens. Diabetes Care.

[15] Kirchner LF, Löhr SS, Guimarães ATB (2012). Avaliação das habilidades sociais de mães de crianças em tratamento de doenças onco-hematológicas. Rev bras crescimento desenvolv hum.

[16] Hauser ST, Jacobson AM, Lavori P, Wolfsdorf JI, Herskowitz RD, Milley JE (1990). Adherence among children and adolescents with insulin-dependent diabetes mellitus over a four-year longitudinal follow-up: II. Immediate and long-term linkages with the family milieu. J Pediatr Psychol.

[17] Stilley CS, Bender CM, Dunbar-Jacob J, Sereika S, Ryan CM (2010). The impact of cognitive function on medication management: three studies. Health Psychol.

[18] Lezak MD (1995). Neuropsychological assessment. 3rd ed..

[19] Hinkin CH, Hardy DJ, Mason KI, Castellon SA, Durvasula RS, Lam MN (2004). Medication adherence in HIV-infected adults: effect of patient age, cognitive status, and substance abuse. AIDS.

[20] Julian LJ, Yelin E, Yazdany J, Panopalis P, Trupin L, Criswell LA (2009). Depression, medication adherence, and service utilization in systemic lupus erythematosus. Arthritis Rheum.

[21] Daleboudt GM, Broadbent E, McQueen F, Kaptein AA (2011). Intentional and unintentional treatment nonadherence in patients with systemic lupus erythematosus. Arthritis Care Res.

[22] Andrade AS, Deutsch R, A Celano S, Duarte NA, Marcotte TD, Umlauf A (2013). Relationships among neurocognitive status, medication adherence measured by pharmacy refill records, and virologic suppression in HIV-infected persons. J Acquir Immune Defic Syndr.

[23] Mellins CA, Brackis-Cott E, Dolezal C, Abrams EJ (2004). The role of psychosocial and family factors in adherence to antiretroviral treatment in human immunodeficiency virus-infected children. Pediatr Infect Dis J.

[24] Bohan A, Peter JB (1975). Polymyositis and dermatomyositis (first of two parts). N Engl J Med.

[25] Hochberg MC (1997). Updating the American College of Rheumatology revised criteria for the classification of systemic lupus erythematosus. Arthritis Rheum.

[26] Petty RE, Southwood TR, Baum J, Bhettay E, Glass DN, Manners P (1998). Revision of the proposed classification criteria for juvenile idiopathic arthritis: Durban, 1997. J Rheumatol.

[27] Petty RE, Southwood TR, Manners P, Baum J, Glass DN, Goldenberg J (2004). International league of associations for rheumatology classification of juvenile idiopathic arthritis: second revision, Edmonton, 2001. J Rheumatol.

[28] Morisky DE, Green LW, Levine DM (1986). Concurrent and predictive validity of a self-reported measure of medication adherence. Med Care.

[29] Teixeira ACA (1998). Adesão ao tratamento farmacológico da hipertensão arterial e seus determinantes em pacientes de ambulatório.

[30] Strelec MAAM, Pierin AMG, Mion D (2003). The influence of patient's consciousness regarding high blood pressure and patient's attitude in face of disease controlling medicine intake. Arq Bras Cardiol.

[31] Varni JW, Quiggens DJL, Ayala GX (2010). Development of the pediatric hematology/oncology parent satisfaction survey. Child Health Care.

[32] Souza FM, Molina J, Terreri MT, Hilario MO, Len CA (2012). Reliability of the pediatric quality of life inventory - healthcare satisfaction generic module 3.0 version for the assessment of the quality of care of children with chronic diseases. J Pediatr.

[33] Smilkstein G (1978). The family APGAR: a proposal for a family function test and its use by physicians. J Fam Pract.

[34] Duarte YAO (2001). Família: rede de suporte ou fator estressor: a ótica de idosos e cuidadores familiares.

[35] Achenbach TM, Rescorla LA (2001). Mental health practitioners guide for the Achenbach system of empirically based assessment (ASEBA). 4th ed.

[36] Lucena-Santos P, Moraes JFD, Oliveira MS (2014). Análise da estrutura fatorial das Escalas Sindrômicas do ASR (Adult Self-Report). Int J Psychol.

[37] Wechsler D (2004). WAIS III - Escala de inteligência Wechsler para adultos - Manual para administração e avaliação.

[38] Coutinho AC (2009). Investigação psicométrica de quatro formas abreviadas do WAIS-III para avaliação da inteligência. [não-publicada].

[39] April KT, Feldman DE, Platt RW, Duffy CM (2006). Comparison between children with juvenile idiopathic arthritis and their parents concerning perceived treatment adherence. Arthritis Rheum.

[40] Degotardi PJ, Revenson TA, Ilowite NT (1999). Family-level coping in juvenile rheumatoid arthritis: assessing the utility of a quantitative family interview. Arthritis Care Res.

[41] Bugni VM, Okamoto KY, Ozaki LS, Teles FM, Molina J, Bueno VC (2011). Development of a questionnaire for early detection of factors associated to the adherence to treatment of children and adolescents with chronic rheumatic diseases — “the pediatric rheumatology adherence questionnaire (PRAQ)”. Arthritis Rheumatoid.

[42] Modi AC, Lim CS, Yu N, Geller D, Wagner MH, Quittner AL (2006). A multi-method assessment of treatment adherence for children with cystic fibrosis. J Cyst Fibros.

[43] Martin S, Elliott-DeSorbo DK, Calabrese S, Wolters PL, Roby G, Brennan T (2009). A comparison of adherence assessment methods utilized in the United States: perspectives of researchers, HIV-infected children, and their caregivers. AIDS Patient Care STDs.

[44] Carter S, Taylor D, Levenson R (2005). A question of choice-compliance in medicine taking. A preliminary review. 2nd ed.

[45] Cano MAT, Ferriani MGC, Gomes R (2000). Sexualidade na adolescência: um estudo bibliográfico. Rev latinoam enferm.

[46] Tosta SP, Carvalho APT (2015). Dinâmicas culturais e educação: apropriação e (re) significação de espaços escolares por adolescentes. Linhas Críticas.

[47] Merzel C, VanDevanter N, Irvine M (2008). Adherence to antiretroviral therapy among older children and adolescents with HIV: a qualitative study of psychosocial contexts. AIDS Patient Care STDs.

[48] Buchanan AL, Montepiedra G, Sirois PA, Kammerer B, Garvie PA, Storm DS (2012). Barriers to medication adherence in HIV-infected children and youth based on self- and caregiver report. Pediatrics.

[49] Lawson EF, Hersh AO, Applebaum MA, Yelin EH, Okumura MJ, von Scheven E (2011). Self-management skills in adolescents with chronic rheumatic disease: a cross-sectional survey. Pediatr Rheumatol Online J.

[50] Lenroot RK, Giedd JN (2006). Brain development in children and adolescents: insights from anatomical magnetic resonance imaging. Neurosci Biobehav Rev.

[51] Tucker LB, Cabral DA (2007). Transition of the adolescent patient with rheumatic disease: issues to consider. Rheum Dis Clin N Am.

[52] Lyketsos CG, Steele C, Galik E, Rosenblatt A, Steinberg M, Warren A (1999). Physical aggression in dementia patients and its relationship to depression. Am J Psychiatry.

[53] Perroud N, Baud P, Mouthon D, Courtet P, Malafosse A (2011). Impulsivity, aggression and suicidal behavior in unipolar and bipolar disorders. J Affect Disord.

[54] Yamazaki S, Sokejima S, Mizoue T, Eboshida A, Fukuhara S (2005). Health-related quality of life of mothers of children with leukemia in Japan. Qual Life Res.

[55] Klassen AF, Klaassen R, Dix D, Pritchard S, Yanofsky R, O'Donnell M (2008). Impact of caring for a child with cancer on parents’ health-related quality of life. J Clin Oncol.

[56] Santo EARE, Gaíva MAM, Espinosa MM, Barbosa DA, Belasco AGS (2011). Taking care of children with cancer: evaluation of the caregivers’ burden and quality of life. Revista Latino-Americana de Enfermagem.

